# Effects of virtual exposure to urban greenways on mental health

**DOI:** 10.3389/fpsyt.2024.1256897

**Published:** 2024-02-22

**Authors:** Xiangrong Jiang, Xiaocan Wang, Linxin He, Qingrui Gu, Xin Wei, Mengfei Xu, William C. Sullivan

**Affiliations:** ^1^ School of Architecture and Civil Engineering, Xihua University, Chengdu, China; ^2^ Department of Landscape Architecture, University of Illinois at Urbana-Champaign, Urbana, IL, United States; ^3^ School of Law and Sociology, Xihua University, Chengdu, China

**Keywords:** urban greenways (UGW), mental health, vegetation design, stress reduction, attention restoration

## Abstract

Urban greenways (UGW) are increasingly recognized as vital components of urban green infrastructure (UGI). While existing research has provided empirical evidence on the positive impacts of UGW on physical health, studies focusing on the effects on mental health remain limited. Moreover, previous investigations predominantly compare UGW as a whole with other built environments, neglecting the influence of specific vegetation designs along UGW on mental health. To address this research gap, we conducted a randomized controlled experiment to examine the impact of vegetation design along UGW on stress reduction and attention restoration. A total of 94 participants were randomly assigned to one of four UGW conditions: grassland, shrubs, grassland and trees, or shrubs and trees. Utilizing immersive virtual reality (VR) technology, participants experienced UGW through a 5-min video presentation. We measured participants’ subjective and objective stress levels and attentional functioning at three time-points: baseline, pre-video watching, and post-video watching. The experimental procedure lasted approximately 40 minutes. Results of the repeated-measures ANOVA revealed that participants experienced increased stress and mental fatigue after the stressor and decreased levels following the UGW intervention. Furthermore, between-group analyses demonstrated that the shrubs group and the grassland and trees group exhibited significantly greater stress reduction than the grassland group. However, there are no significant differences in attention restoration effects between the four groups. In conclusion, virtual exposure to UGW featuring vegetation on both sides positively affected stress reduction and attention restoration. It is recommended that future UGW construction incorporates diverse vegetation designs, including shrubs or trees, instead of solely relying on grassland. More research is needed to explore the combined effects of shrubs and trees on mental health outcomes.

## 1 Introduction

Rapid and dense urbanization has occurred around the world, especially in developing nations (1). The grievous degradation of the urban environment has created an urgent need for green space interventions (2), while people have fewer chances to contact nature (3). The loss of urban nature can exacerbate air pollution, traffic noise, and sedentary routines, negatively affecting the physical and mental health of urban residents (4–6). In the meantime, city administrators, academics, and the public have already acknowledged urban nature’s value in promoting public health. The top motivations for urban residents to visit urban nature are exercising and escaping the tension of city life (7), It is believed that exposure to urban nature could facilitate residents’ healthy behaviors (8, 9), reduce stress (10, 11), and promote social interactions (12, 13), thereby providing long-term health benefits at the population level (14, 15).

The aforementioned evidence aligns with theories in the field of environmental psychology, such as Attention Restoration Theory (16) and Stress Reduction Theory (17, 18). Attention Restoration Theory (ART) places significant emphasis on the capacity of natural environments to replenish cognitive functions that become fatigued or depleted during tasks requiring directed attention. As per the ART framework, a restorative environment ought to simulate an escape from one’s daily routine, provide “soft fascination” that effortlessly draws attention, offer a sense of extent that is rich and coherent enough to engage the mind and align with the individual’s goals (19). Similarly, Ulrich’s Stress Reduction Theory (SRT) posits that the mere presence of nature has the potential to mitigate tension and enhance one’s general state of well-being (10, 11). This underscores the capacity of natural environments to exert a beneficial influence on both physiological and psychological stress. By applying these theoretical frameworks of ART and SRT to the utilization of urban nature for promoting public health, it becomes possible to suggest that deliberately planned urban green spaces could function as beneficial assets in terms of mental health. Building on the principles of ART and SRT, researchers have demonstrated to what extent exposure to various types of nature may promote public health (5, 20, 21). These natural resources with different characteristics (e.g. tree canopy density, vegetation diversity, etc.) can offer opportunities for individuals to engage with nature, fostering attention restoration and stress reduction amid the demands of city life.

Given the significant influence of urban nature on public health, urban green infrastructure (UGI), which serves as the primary source of nature for urban residents, has garnered increasing attention from administrators and academics (21–23). UGI, including various urban nature types, such as parks, green spaces, and urban forests, has been widely recognized for its significant impacts on promoting public health (23–25). These urban green spaces provide residents opportunities for physical activity (8), relaxation (26), and social interaction (27), all of which contribute to improved physical and mental health. Urban greenways (UGW), which are linear open green spaces of UGI, are an important type of UGI for recreation and transportation around the world. UGW as landscaped and traffic-calm corridors for pedestrians and cyclists frequently connect urban green spaces and other open spaces (28, 29), and is increasingly recognized as an effective resource for promoting public health. Building or retrofitting UGW has been identified as one of the most effective UGI intervention strategies for cities to improve public health in cities (23, 30, 31).

Many researchers have already demonstrated empirical evidence of the advantages of having access to UGW (32–34). Substantial evidence suggests that UGW can provide opportunities for physical activity, such as walking, cycling, and jogging, which can reduce the risk of obesity (35), promote cardiovascular health, and decrease the prevalence of chronic diseases such as diabetes and hypertension (36). Greenway intervention is believed to promote physical health effectively (8, 36, 37). Specifically, researchers discovered that the greenway intervention had a positive effect on people’s physical activity levels after its construction by comparing data collected before and after the construction of a UGW in Wuhan (8). Using questionnaires and an Importance-Performance Analysis (IPA) model, Xu, Shi (32) demonstrated that UGW can facilitate physical activity, thereby promoting healthy lifestyles. Price, Reed (33) surveyed 1,148 greenway users and found that 91% of respondents engaged in physical activity on the greenway, suggesting that greenways can promote physiological health by encouraging physical activity. In another study, researchers developed a macro-simulation model to assess the impact of a greenway intervention in Northern Ireland. The results suggest that greenways can increase people’s activity levels, which ultimately leads to improved physical health (36). By providing safe and easily accessible paths for physical activity, UGW can encourage regular exercise, which has been shown to have positive impacts on physical health.

Besides the promotion of physical activities, the intervention of UGW can also contribute to improvement of mental health. Hartig, Mang (38) conducted an experiment involving three categories of participants. After completing a mental fatigue-inducing task, one group of participants walked in a natural environment, another group walked in an urban environment, and a third group listened to light music and read magazines. Individuals who walked in a natural environment exhibited greater psychological restoration and cognitive performance, according to the findings. Walking in a forest path is believed to produce consistent improvements in psychological state (39). In another study, participants who walked in nature had better attentional functioning than their counterparts who walked in urban built environments (26). In these studies, wandering in a natural setting is comparable to the experience of visiting an urban greenway, which provides recreation and relaxation destinations. However, it is important to note that most of the research on mental health-promoting outcomes focuses on the general urban green infrastructure (UGI), which encompasses a diverse variety of categories and characteristics. For example, accumulated evidence supports the positive impact on mental health by exposure to UGI (21, 23, 40, 41). Previous research supports the effects of UGI on reducing stress and anxiety (10, 42), enhancing mood (5, 43, 44) and improving cognitive function (21, 45). Researchers have also investigated specific characteristics of UGI, such as vegetation density (10), biodiversity (42), vertical diversity of vegetation design (20) etc., that may impact health outcomes. These findings suggest that the quality and features of UGI are also an important determinant of its health-promoting benefits.

Although there is cumulative evidence for the beneficial effects of UGI on health outcomes, our comprehension of the effects of different types of UGI vary considerably. Previous studies often focus on certain types of UGI, such as street trees and parks, for mental health benefits, while we still know little about the impacts of UGW on mental health. Research on impacts of UGW mainly focuses on promoting physical activity and physical health, and few studies have explored the benefits of mental health by exposure to UGW. Moreover, previous studies often regard UGW as a whole entity or just as a type of UGI. Few studies have delved into exploring the natural characteristics or quality of these linear green spaces extensively. We still know little about the impacts of vegetation design of UGW on mental health. The gap in our knowledge may prevent us from optimizing the design of vegetation along UGW, thereby compromising the anticipated health benefits. Therefore, this paper investigates different varieties of vegetation design on both sides of UGW and their impacts on mental health.

In our study, we have conducted a controlled experiment to explore the impact of vegetation design along UGW on people’s mental health. Our hypothesis is that variation of vegetation design along UGW may contribute differently to mental health. Using virtual reality (VR) technology, participants were randomly assigned to one of four groups to observe UGW with various vegetation designs. Next, we report both subjective and objective measures of the mental health of the participants, and we end by discussing the implications for UGW design.

## 2 Materials and methods

### 2.1 Materials

This study investigated the impacts of exposure to UGW with different vegetation designs on mental health outcomes in Chengdu, China. We chose the Tianfu Greenway as our study area because it is a recently constructed urban green infrastructure, which is intended to provide the public with urban nature and open spaces. The Tianfu Greenway serves as a ring trail along the Bypass Expressway in Chengdu. It is also the preeminent greenway that links over a hundred urban eco-parks in the city. While the Tianfu Greenway circumnavigates the city rather than being situated within a densely populated urban area, its primary user base comprises urbanites seeking a sanctuary from the urban built environments. As a nearby destination for exploring outdoors, the Tianfu Greenway is also not too far from the doorsteps of urban residents. Since opening to the public in 2022, the 100-kilometer-long Tianfu Greenway has become a popular destination for urban residents.

### 2.2 Virtual exposure to greenways

**Figure 1:**
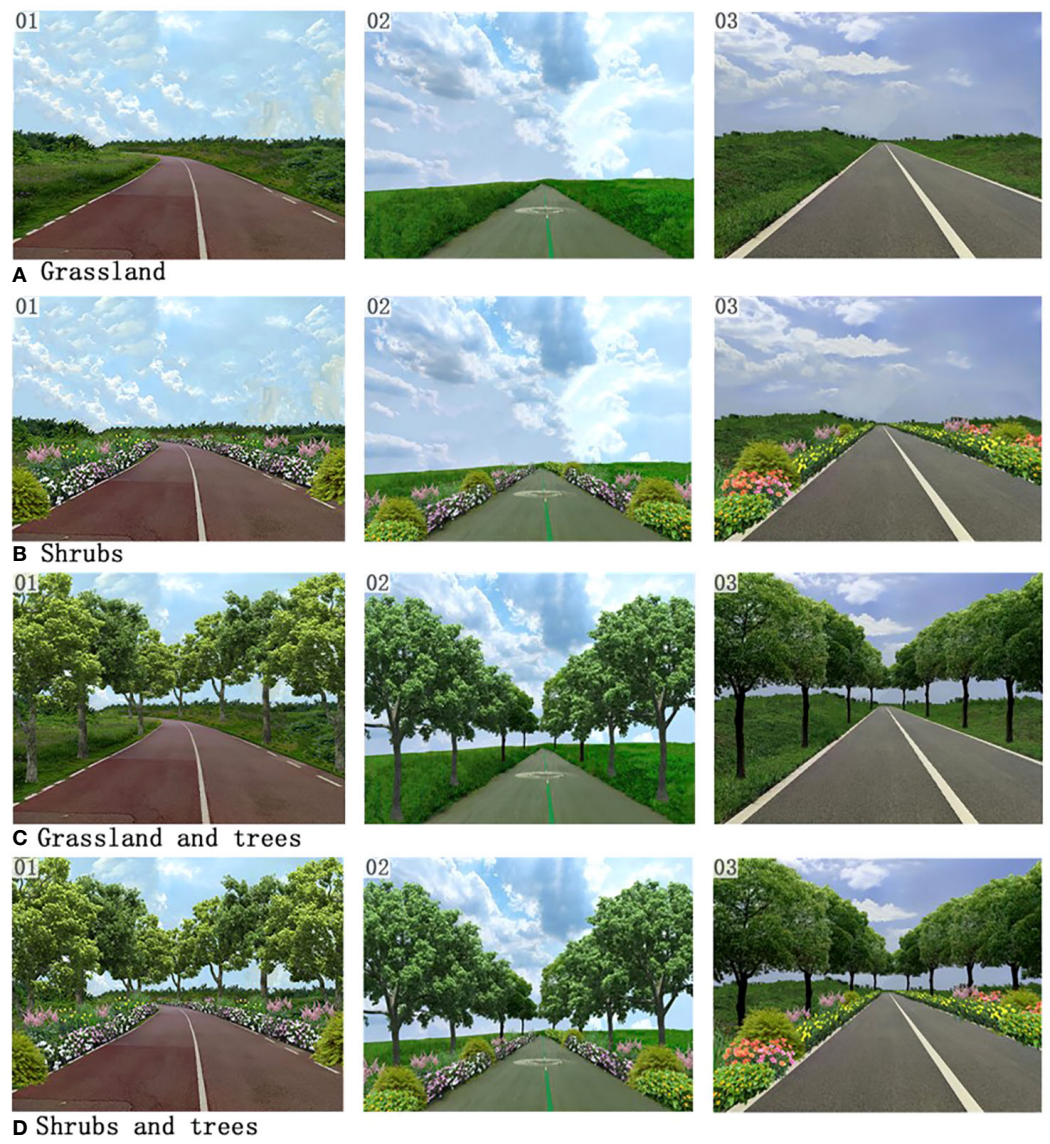
Example images from the Grassland **(A)**, Shrubs **(B)**, Grassland and trees **(C)**, and Shrubs and trees **(D)** groups.

We took pictures along the Tianfu Greenway at approximately 160 cm above the ground between 10:00 a.m. and 3:00 p.m. on sunny days in summer and avoided including any buildings or other man-made structures. Those inevitable non-natural elements, such as buildings or man-made structures, in the images have been removed by Photoshop to avoid confounding variables. We aimed to isolate the effects of vegetation design along UGW on mental health. The focal point of each image was the path and adjacent green spaces. Then, we selected 15 images containing only the path and grassland, thereby creating the first category: grassland. Next, we added shrubs and flowers using Adobe Photoshop to produce the second category: shrubs. Then, we added trees to the first category and established a third category called grassland and trees. Finally, we added trees to the second category, resulting in the fourth category: shrubs and trees (**Figure 1**). In every picture, we kept the distant trees in the background as we wanted to demonstrate the natural surroundings of UGW. We made an effort to select vegetation species that are prevalent along the Tianfu Greenway. Therefore, the images we used in this study are representative examples of urban greenways for the local participants. As shown in **Figure 1**, the Tianfu Greenway is more like a landscaped urban trial within urban fabric instead of a forest-based natural trail. Using Adobe Premiere Pro, we created a five-minute video for each group based on the 15 images depicting various vegetation designs. Each image in the four videos would last 20 seconds.

In this study, we employed VR to investigate the impact of urban greenways on mental health because VR can provide a controlled and highly immersive environment that closely replicates real-world settings, enhancing the external validity of our study. We only produced a five-minute video with those images to prevent participants from experiencing nausea and headache as a result of wearing the VR headset. The videos are not 360-degrees but are shot from a similar perspective as when people are strolling along a greenway. We employed VIVE-2Q2R100 of the VIVE Cosmos series with 3-degrees of freedom in the study. Participants donning the virtual reality headset could turn their heads from side to side but not above the shoulders to observe each image with a field of view not greater than 110°. Six camera sensors in the virtual reality headset work in conjunction with software-optimized features to provide accurate inside-out tracking. The device’s combined pixel resolution of 2880 * 1700 and refresh rate of 90 Hz allow participants to view VR videos with excellent visual clarity and easy data transfer.

### 2.3 Participants

University students who are at least 17 years old and have normal hearing and vision were eligible to participate. Our selection of university students as the sample population for this research was deliberate and predicted on several factors. First, university students are a demographic often experiencing stress and mental fatigue due to academic pressures, making them a relevant population for investigating the potential restorative effects of urban greenways. Second, university students typically process greater technological proficiency, including VR headsets, which ensures a streamlined and uniform experience during the virtual exposure to UGW. Individuals who self-reported a history of cardiovascular disease, depression, or post-traumatic stress disorder were excluded. We validated the inclusion and exclusion criteria using a health check form in the recruiting material which we posted on a volunteer platform shared by multiple universities in Chengdu. Volunteer credits would be granted to students who participated in the experiment. We aimed to recruit about 100 university students to participate in our study. Additionally, we instructed participants not to consume caffeinated foods within 24 hours prior to the experiment. Prior to the beginning of the experiment, participants were given a summary of the research and the experimental procedure. They were also asked to sign the consent form.

### 2.4 Experimental procedures

**Figure 2:**
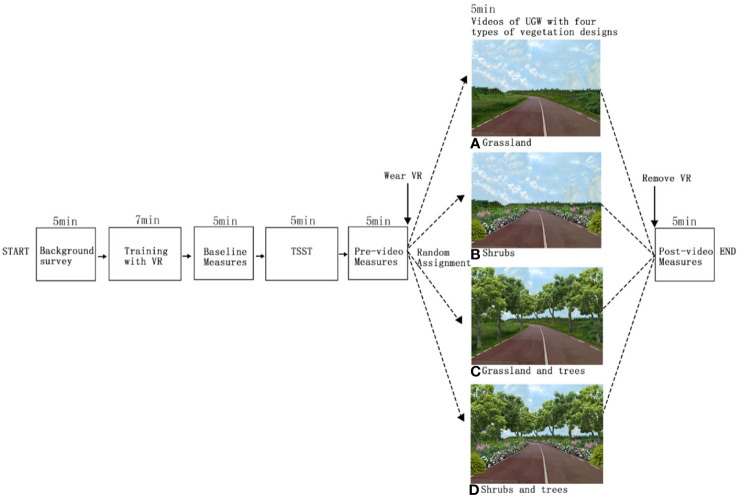
Experimental procedures. Labels of **(A–D)** indicate one of the four groups participants might be assigned to.

Participants showed up at their assigned time. Lab staff explained the potential risks associated with the experimental procedures. We ensured that each participant was aware that they could quit the experiment at any moment if they felt uncomfortable. After signing the consent form, participants rested for 5 minutes in the reception area. Then, participants completed the background survey. Afterwards, participants entered the lab and received training for wearing the VR headset. The experiment progressed through the following steps (**Figure 2**):

**Step 1: baseline measures.** Participants completed the Profile of Mood States (POMS) questionnaire (46) to assess their level of stress and the Visual Analog Scale (VAS) to assess their attentional functioning. Lab staff measured participants’ systolic blood pressure (SBP), diastolic blood pressure (DBP), and heart rate (HR) with the Omron T30J electronic sphygmomanometer. Participants then took the digit span backward (DB) tests.
**Step 2: Trier Social Stress Test (TSST).** The TSST was used to stress out the participants. Lab staff began by inquiring about the participants’ post-graduation plans (e.g. graduate school, employment, etc.). After answering the question, participants were given 30 seconds to compose a 2-minute speech to get their ideal offer. During this time, lab staffs did not provide any feedback. Next, participants were instructed to subtract 13 from 1022, followed by subtracting another 13 from the result. Participants continued doing so until they made a mistake, which caused them to return to subtracting 13 from 1022. The duration of the mental arithmetic is one minute.
**Step 3: pre-video measures.** After TSST, participants would retake the POMS, VAS, and DB, as well as have their SBP, DBP, and HR measured.
**Step 4: video watching.** In this laboratory experiment, lab staff assisted participants wearing the VR headset to simulate exposure to UGW. During the five-minute duration of the video, participants may move their heads from side to side but not over their shoulders to view UGW.
**Step 5: post-video measures.** Lab staff helped participants remove the VR headset. The participants would complete the POMS, VAS, and DB for the final time, and their SBP, DBP, and HR would be measured once more.

After completing the preceding procedures, participants walked out the laboratory.

### 2.5 Construct and measures

The objectives of this study were to investigate the impact of vegetation design along UGW on people’s mental health. The independent variable was video-based virtual exposure to UGW with different vegetation design. The dependent variables include indicators of participants’ stress level and attentional functioning for the participants.

We designed a controlled experiment to examine the effects of virtual exposure to UGW with four distinct vegetation designs, which provides an opportunity for the participants to experience the restorative effects. The psychological restoration discussed in this study refers to the process by which exposure to UGW contributes to the recovery of stress and attentional functioning. The concept pf psychological restoration is deeply rooted in environmental psychology and has been extensively explored in the context of ART and SRT (10, 11, 47, 48). As the consensus has been reached regarding the psychological benefits of nature’s restorative properties, researchers have begun to investigate which natural features and species provide the ideal restorative advantages (5, 20, 21).

To investigate the restorative effects of UGW, we first increased their stress level and then fatigued the participants. In an experiment with a within-subject research design, the optimal effect on stress increase and mental fatigue could be achieved by exposing each participant to the stressor only once, thereby avoiding the possibility of a carry-over effect. Following the stressor, participants were randomly assigned to one of the four groups and viewed the UGW with a VR headset. Therefore, participants can experience psychological restoration in the status of stress and mental fatigue.

**Figure 3:**
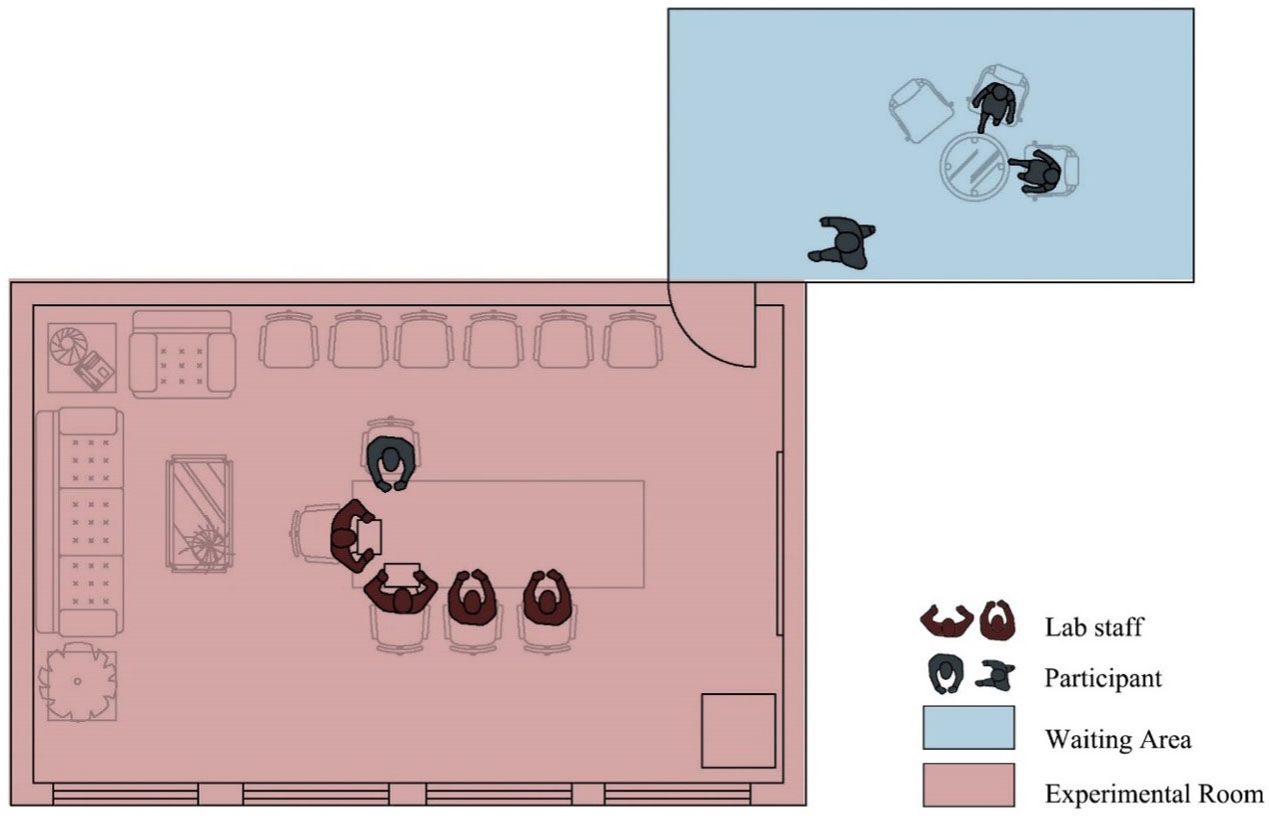
Plan of the lab and placement of participants and lab staffs during the experiment.

We measured the subjective and objective stress levels and attentional functioning of the participants three times, at baseline, before and after viewing the video. The experiment was conducted in a room with closed curtains and a waiting area outside for approximately forty minutes (**Figure 3**). We expected to find variance in stress and attention across the three measurements within each group. Meanwhile, we also wanted to identify differences between groups as a consequence of their exposure to UGW with varying vegetation designs.

#### 2.5.1 Indicators of stress levels

The research measured both the objective and subjective stress levels of participants. We measured the objective stress level of participants using physiological indicators such as blood pressure (SBP and DBP) and HR. Our laboratory personnel assisted participants in wearing the Omron sphygmomanometer and recording the raw data.

For the subjective stress measurement, we employed POMS – a questionnaire that typically consists of a series of adjectives or phrases that characterize different mood states, such as tension, anger, depression, fatigue, and confusion. On a 6-point scale, participants were asked to rate the extent to which each mood state applies to them in the past week. POMS is widely used in research and clinical contexts to measure changes in mood state over time or in response to a specific intervention (49). In this study, participants were rated as more stressed when their POMS scores were larger. Overall, higher values of SBP, DBP, HR, and POMS indicated increased levels of stress.

#### 2.5.2 Indicators of attentional functioning

Similarly, we measured both objective and subjective attention of participants. The digit span backward (DB) was employed to assess participants’ objective attentional functioning (50). The rationale for this choice is grounded in the understanding that tasks requiring the backward recall of digits necessitate sustained attention to manipulate and reorder the presented information. In the DB tests, participants heard a series of digits and were requested to recite them in the opposite order. For example, the correct response would be “8, 2, 5” if the digits “5, 2, 8” were presented. The DB tests required participants to repeat sequences of increasing lengths of digits until failing twice in a row. The tests have been used in previous studies to assess attentional capacity, short-term memory and working memory (51, 52). The interdependence between attentional functions and working memory tasks is well-established. Engle, Tuholski (53) explored the relationship between working memory, short-term memory, and fluid intelligence, highlighting the attentional demands inherent in working memory tasks. Models of working memory have been proposed to underscore the central role of attention for the activation and maintenance of information in working memory (54, 55). DB is believed to associate with attention and executive function processes (56). Participants’ ability to correctly recite digits was recorded and utilized in the subsequent statistical analysis. The more numerals participants can recall, the better their attention.

For the subjective measurement of attention, we employed the Visual Analog Scale (VAS), a subjective rating scale commonly used in research and clinical settings to assess the intensity or magnitude of subjective experiences such as pain, anxiety, mood, or satisfaction. Participants were asked to rate their extent of mental fatigue by placing a mark on a line representing their level of intensity, with the distance from the start of the line being used to quantify the rating. We inverted the VAS scores, so that larger values on the VAS indicated better attentional functioning, aligning with the order of the DB scores. For the indicators of attention, the larger values represent better attentional functioning.

## 3 Results

**Figure 4:**
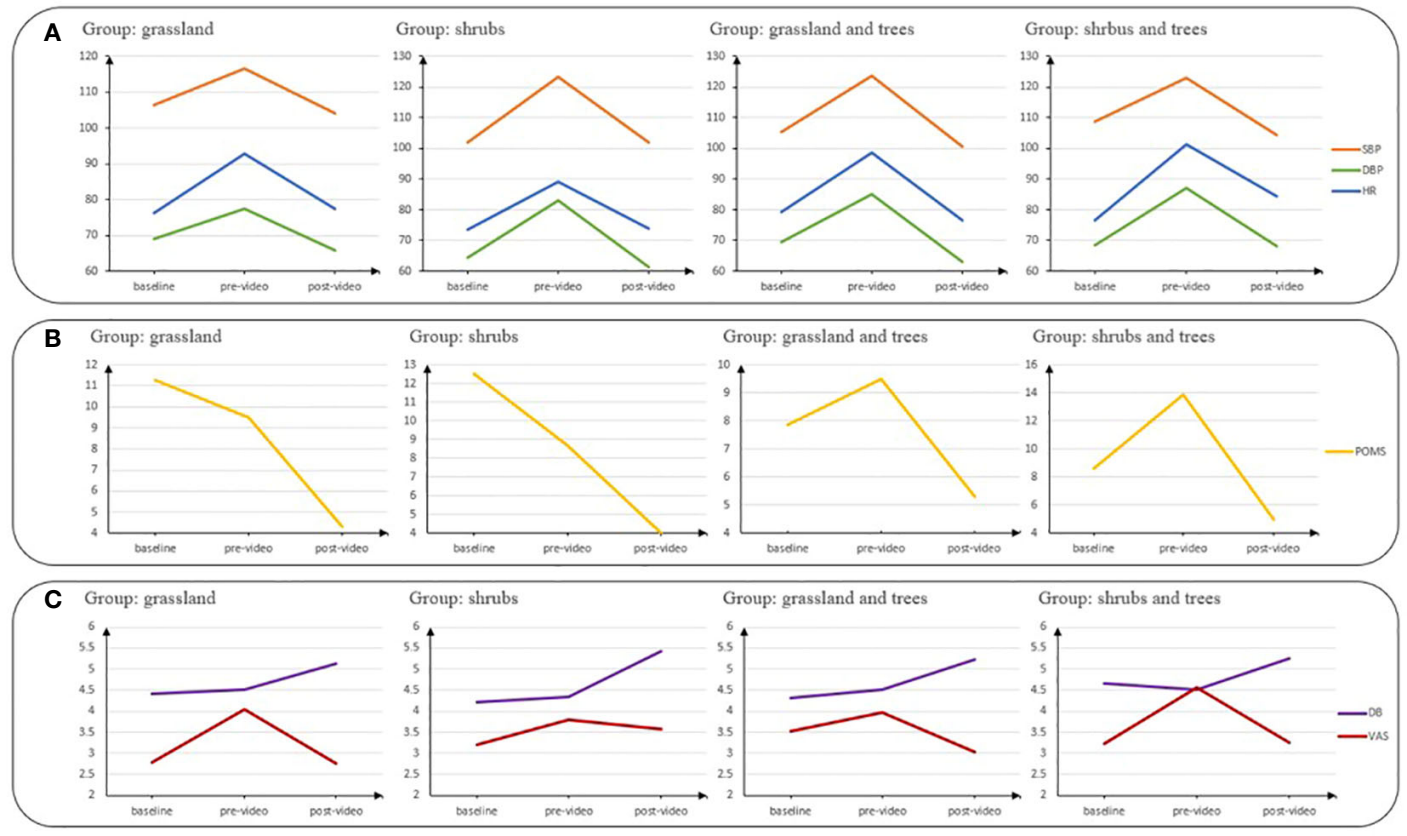
Variations in stress and attention indicators across the four groups with different vegetation designs. Row **(A)** presents the distributions of objective stress levels measured by systolic blood pressure (SBP), diastolic blood pressure (DBP), and heart rate (HR), while row **(B)** displays the subjective stress level measured by POMS. Row **(C)** represents attentional functioning assessed by Digit Span Backwards (DB) and subjective mental fatigue reported on a Visual Analog Scale (VAS). The four groups include grassland, shrubs, grassland and trees, and shrubs and trees.

Some potential participants faced health-related issues during the experiment period, impacting their ability to participate. As a result, the study was conducted with 94 participants. We randomly assigned each participant to watch one of the four videos. 24 participants were assigned to the grassland group, 24 to the shrubs group, 23 to the grassland and trees group, and 23 to the shrubs and trees group. According to the background survey, participants’ ages range between 18 and 26 years old. There is no significant difference in age (p = 0.68) or gender (p = 0.81) among the four groups (**Table 1**). Objective and subjective measures were used to assess participants’ stress levels and attentional functioning in the study. The descriptive statistics of stress and attention indicators are reported in **Table 2**. **Figure 4** further visually depicts the pre- and post-averages of these indicators across the four groups.

**Table 1:** Demographic statistics of age in the four groups.

	Age						Gender	
Groups	No.	Mean Age	Std. Deviation	Std. Error	Minimum	Maximum	Male	Female
grassland	24	19.58	1.56	0.32	18	23	6	17
shrubs	24	19.54	2.52	0.51	18	25	5	19
grassland and trees	23	19.26	1.98	0.41	18	26	5	19
shrubs and trees	23	19.00	0.95	0.20	18	21	4	19
Total	94	19.35	1.84	0.19	18	26	20	74

**Table 2:** Descriptive statistics of stress and attention indicators in four groups.

measures		number	baseline	pre-video	post-video
			M ± SD	M ± SD	M ± SD
systolic blood pressure (SBP)	grassland	24	106.37 ± 14.90	116.71 ± 19.84	104.17 ± 13.97
	shrubs	24	102.00 ± 10.82	123.25 ± 13.27	101.98 ± 12.93
	grassland and trees	23	105.43 ± 9.68	123.57 ± 14.32	100.61 ± 8.50
	shrubs and trees	23	108.83 ± 12.53	123.09 ± 10.82	104.48 ± 11.33
diastolic blood pressure (DBP)	grassland	24	69.13 ± 13.28	77.42 ± 13.33	65.83 ± 10.27
	shrubs	24	64.29 ± 8.56	82.92 ± 10.97	61.50 ± 8.52
	grassland and trees	23	69.61 ± 12.81	85.09 ± 12.46	63.04 ± 8.25
	shrubs and trees	23	68.48 ± 10.31	87.00 ± 14.65	68.13 ± 10.37
heart rate (HR)	grassland	24	76.21 ± 9.21	92.83 ± 15.68	77.42 ± 12.37
	shrubs	24	73.46 ± 9.69	89.08 ± 17.54	74.04 ± 9.47
	grassland and trees	23	79.35 ± 15.57	98.52 ± 22.16	76.43 ± 14.70
	shrubs and trees	23	76.57 ± 10.79	101.35 ± 22.95	84.43 ± 24.61
profile of mood states (POMS)	grassland	24	11.29 ± 10.76	9.50 ± 5.53	4.33 ± 6.46
	shrubs	24	12.50 ± 12.00	8.67 ± 6.92	4.04 ± 6.59
	grassland and trees	23	7.87 ± 9.96	9.48 ± 5.46	5.30 ± 6.57
	shrubs and trees	23	8.65 ± 8.71	13.87 ± 10.11	4.96 ± 7.80
digit span backward (DB)	grassland	24	4.42 ± 1.44	4.54 ± 1.25	5.13 ± 1.65
	shrubs	24	4.21 ± 1.29	4.33 ± 1.20	5.42 ± 1.72
	grassland and trees	23	4.30 ± 1.80	4.52 ± 1.68	5.22 ± 1.81
	shrubs and trees	23	4.65 ± 1.75	4.52 ± 1.28	5.26 ± 1.45
visual analog scale (VAS)	grassland	24	2.79 ± 1.79	.04 ± 1.78	2.75 ± 2.03
	shrubs	24	3.21 ± 1.89	3.79 ± 2.21	2.58 ± 1.61
	grassland and trees	23	3.52 ± 2.13	3.96 ± 2.21	3.04 ± 1.72
	shrubs and trees	23	3.22 ± 1.73	4.57 ± 1.73	3.26 ± 1.32

After exploring the data distribution of indicators representing stress and attention, we found that all indicators were normally distributed except HR. The Z-scores of data representing HR in the normality test (Shapiro-Wilk test) were larger than 1.96, which suggests variable of HR is not normally distributed. Based on commonly accepted statistical criteria, outliers are typically characterized by Z-scores beyond ±2, and extreme outliers are those beyond ±3. In the three measurements of HR, the baseline measurement contained two outliers, while the post-video measurement contained four outliers. Among these outliers, two extremes exist in the datasets of HR. In the following analysis, we applied ANOVA to explore datasets of all indicators except those of HR. As the dataset of HR is not normally distributed, we applied the nonparametric test (Mann-Whitney U test) which does not assume normality. The Mann-Whitney U test is robust to outliers and better suited for datasets with non-normally distributed variables. Another reason for us to keep the outliers is that extreme values in HR were not data collection errors but rather reflected a meaningful physiological response. The observed pattern in HR outliers aligns with expectations during the experiment, where the baseline measure is followed by an initial increase after TSST and a decrease after the video intervention. Therefore, we have chosen to retain outliers in the dataset of HR to explore their potential impact on our research questions.

We continued to compare the indicators of stress and attention at baseline among the four groups. As can be seen in **Table 3**, there are no significant differences in the baseline measures of stress or attention among the four groups. Similarly, there is no significant difference in the baseline measure of HR (p=0.71).

**Table 3:** One-way ANOVA of stress and attention in the four groups at baseline measures.

Baseline measures		Sum of Squares		df	Mean Square	F	Sig.
Objective stress level	systolic blood pressure (SBP)	Between Groups	565.39	3	188.46	1.27	.29
		Within Groups	13308.58	90	147.87		
		Total	13873.97	93			
	diastolic blood pressure (DBP)	Between Groups	423.11	3	141.04	1.09	.36
		Within Groups	11690.80	90	129.90		
		Total	12113.92	93			
Subjective stress level	profile of mood states (POMS)	Between Groups	334.93	3	111.64	1.02	.39
		Within Groups	9820.79	90	109.12		
		Total	10155.71	93			
Objective attentional functioning	digit span backward (DB)	Between Groups	2.55	3	.85	.34	.79
		Within Groups	223.88	90	2.49		
		Total	226.44	93			
Subjective attentional functioning	visual analog scale (VAS)	Between Groups	6.36	3	2.12	.59	.62
		Within Groups	321.57	90	3.57		
		Total	327.93	93			

### 3.1 Variation of stress and attention between baseline, pre- and post-videos measures

We assessed the mental fatigue induced by TSST and the attention restoration promoted by the video intervention with repeated-measures ANOVA. The analysis included video treatment as the between-subject factor and time as the within-subject factor.

#### 3.1.1 Variation of stress levels

In the analysis of repeated-measures ANOVA for stress indicators, as the tests of sphericity were violated and epsilon (ϵ) is larger than 0.75, we applied the Huynh-Feldt correction (57). The results revealed that there was a significant variation in these three stress indicators – SBP, DBP and POMS (**Table 4**). Both objective and subjective measurements of stress varied significantly across measurements at the three-time points, which suggest that TSST introduced acute stress to participants, and the virtual exposure to UGW managed to reduce stress.

**Table 4:** Repeated-measures ANOVA of stress indicators.

stress indicators	source	df	F	Sig.	Partial Eta Squared
systolic blood pressure (SBP)	time	1.70	147.41	<0.001	0.62
	time*group	5.09	2.87	0.02	0.09
diastolic blood pressure (DBP)	time	1.91	156.69	<0.001	0.64
	time*group	5.73	3.60	0.00	0.11
profile of mood states (POMS)	time	1.84	25.43	<0.001	0.22
	time*group	5.50	2.86	0.01	0.09

Specifically, TSST increased participants’ objective measures of stress (SBP and DBP), but their subjective stress level (POMS) stayed steady from the baseline measure to the pre-video measure. The video intervention decreased both objective and subjective measures of stress, which suggests that virtual exposure to greenway has a significant effect on stress reduction. To further investigate the stress-reducing effect of UGW, we conducted pairwise comparisons of stress levels at the baseline, pre-video, and post-video (**Table 5**). Results indicate that stress indicators including SBP, DBP, and POMS of the post-video measures were lower than the counterparts at the pre-video as well as those at the baseline.

**Table 5:** Pairwise comparisons of stress indicators at the baseline, pre-video and post-video.

Measure	(I) time	(J) time	Mean Difference (I-J)	Std. Error	Sig.^b^	95% Confidence Interval for Difference^b^	
						Lower Bound	Upper Bound
systolic blood pressure (SBP)	baseline	pre-video	-15.99^*^	1.34	<0.001	-19.27	-12.72
		post-video	3.08^*^	0.86	0.002	0.99	5.16
	pre-video	baseline	15.99^*^	1.34	<0.001	12.72	19.27
		post-video	19.07^*^	1.32	<0.001	15.86	22.28
diastolic blood pressure (DBP)	baseline	pre-video	-15.23^*^	1.28	<0.001	-18.35	-12.11
		post-video	3.25^*^	0.99	0.005	0.83	5.67
	pre-video	baseline	15.23^*^	1.28	<0.001	12.11	18.35
		post-video	18.48^*^	1.05	<0.001	15.91	21.04
profile of mood states (POMS)	baseline	pre-video	-0.3	1.00	1	-2.75	2.14
		post-video	5.42^*^	0.97	<0.001	3.06	7.78
	pre-video	baseline	0.3	1.00	1	-2.14	2.75
		post-video	5.72^*^	0.71	<0.001	3.99	7.44

*The mean difference is significant at the .05 level.

^b.^Adjustment for multiple comparisons: Bonferroni.

In light of the non-normal distribution of the HR data, the Mann-Whitney U test was implemented. Results indicate that there was not significant difference of HR between the baseline and the post-video measures, while HR at the post-video was different from that at the pre-video. These results suggest that virtual exposure to UGW can reduce acute stress, as measured subjectively and objectively.

#### 3.1.2 Repeated-measures ANOVA for attentional functioning

In the analysis of repeated-measures ANOVA for attention indicators, the tests of sphericity were passed, and we reported the results of Pillai’s Trace. The results revealed that there was significant variation in both objective and subjective attention indicators. Participants’ attention was fatigued after the TSST and restored after the video intervention (**Table 6**).

**Table 6:** Repeated-measures ANOVA of attention indicators.

measures	source	df	F	p	Partial Eta Squared
digit span backward (DB)	time	2	23.17	<0.001	0.34
	time*group	6	0.66	0.68	0.02
visual analog scale (VAS)	time	2	41.62	<0.001	0.48
	time*group	6	1.22	0.3	0.04

Specifically, TSST did fatigue participants subjectively (**Table 7**). The value of VAS decreased from the baseline to the pre-video. However, the results of DB stay the same between the first two measurements. Moreover, virtual exposure to UGW can restore participants’ mental fatigue, both objectively and subjectively. According to the results of DB, participants’ attentional functioning by the end of the video intervention was not only better than that at the pre-video but also better than that at the baseline. Though the subjective measure of attention did not differ significantly between the baseline and the post-video measure, the result of VAS showed significant attention restoration between the pre-video and post-video measures.

**Table 7:** Pairwise Comparison of Attentional Functioning.

Measure	(I) time	(J) time	Mean Difference (I-J)	Std. Error	Sig.^b^	95% Confidence Interval for Difference^b^	
						Lower Bound	Upper Bound
digit span backward (DB)	baseline	pre-video	-0.08	0.12	1	-0.37	0.20
		post-video	-0.86^*^	0.14	<0.001	-1.19	-0.53
	pre-video	baseline	0.08	0.12	1	-0.20	0.37
		post-video	-0.78^*^	0.13	<0.001	-1.09	-0.46
visual analog scale (VAS)	baseline	pre-video	0.90^*^	0.15	<0.001	0.53	1.28
		post-video	-0.28	0.14	0.17	-0.62	0.07
	pre-video	baseline	-0.90^*^	0.15	<0.001	-1.28	-0.53
		post-video	-1.18^*^	0.13	<0.001	-1.50	-0.86

*The mean difference is significant at the .05 level.

^b^Adjustment for multiple comparisons: Bonferroni.

### 3.2 Differences in restorative effects among groups

We examined the differences in the impact of video intervention on recovery from stress and mental fatigue among the four groups. First, we started with ANCOVA with video interventions as the between-subject factor and gender as the covariate. The results suggest that the effect of video interventions on indicators of stress and attention is consistent across different genders. Then, we employed a one-way ANOVA with video intervention as the between-subject factor to compare the difference between pre-video and post-video measures of stress and attention. We found significant between-group differences for SBP and DBP (**Table 8**). For SBP, the effect size (partial η^2^) is 0.1, while the effect size for DBP is 0.15. The other stress indicator – POMS, as well as both attention indicators are not significantly different between groups.

**Table 8:** One-way ANOVA of difference between pre-video and post-video measures of stress indicators.

		Sum of Squares	df	Mean Square	F	Sig.
systolic blood pressure (SBP)	Between Groups	1605.18	3.00	535.06	3.29	0.02
	Within Groups	14645.73	90.00	162.73		
	Total	16250.90	93.00			
diastolic blood pressure (DBP)	Between Groups	1643.89	3.00	547.96	5.28	0.00
	Within Groups	9347.23	90.00	103.86		
	Total	10991.12	93.00			

**Figure 5:**
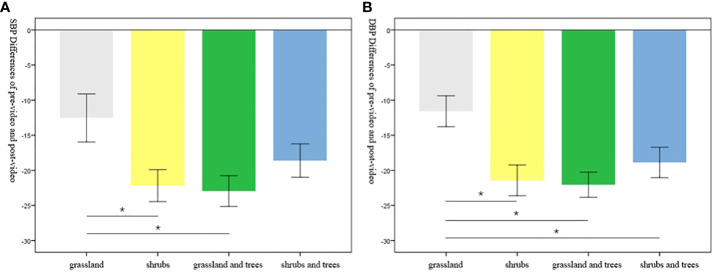
The differences in SBP **(A)** and DBP **(B)** between pre-video and post-video across the four groups. Error bars represent variations of indicators with a 95% Confidence Interval (CI). * indicates significant difference.

To examine the extent to which type of UGW can promote better stress reduction, we made pairwise comparisons using LSD (**Table 9**). The basic values for the *post-hoc* tests are reported in **Table 10**. The difference in SBP between pre-video and post-video demonstrates that participants in the grassland group had less stress recovery compared to their counterparts in the shrubs group and grassland and trees group. The difference in DBP between pre-video and post-video demonstrates that participants in the grassland group had less stress recovery compared to their counterparts in the other three groups (**Figure 5**). Overall, participants exposed to UGW with grassland had less reduction in blood pressure, which represented less stress reduction.

**Table 9:** *Post-hoc* tests of the difference of pre-video and post-video tests on stress indicators.

1= grassland; 2=shrubs; 3= grassland and trees; 4=shrubs and trees;			Mean Difference of pre-video and post-video tests	Std. Error	Sig.	95% Confidence Interval	
						Lower Bound	Upper Bound
systolic blood pressure (SBP)	1	2	9.63^*^	3.68	.01	2.31	16.94
		3	10.42^*^	3.72	.01	3.02	17.81
		4	6.07	3.72	.11	-1.33	13.46
	2	1	-9.63^*^	3.68	.01	-16.94	-2.31
		3	.79	3.72	.83	-6.61	8.18
		4	-3.56	3.72	.34	-10.95	3.84
	3	1	-10.42^*^	3.72	.01	-17.81	-3.02
		2	-.79	3.72	.83	-8.18	6.61
		4	-4.35	3.76	.25	-11.82	3.13
	4	1	-6.07	3.72	.11	-13.46	1.33
		2	3.56	3.72	.34	-3.84	10.95
		3	4.35	3.76	.25	-3.13	11.82
diastolic blood pressure (DBP)	1	2	9.83^*^	2.94	<0.01	3.99	15.68
		3	10.46^*^	2.97	<0.01	4.55	16.37
		4	7.29^*^	2.97	0.02	1.38	13.19
	2	1	-9.83^*^	2.94	<0.01	-15.68	-3.99
		3	0.63	2.97	0.83	-5.28	6.53
		4	-2.55	2.97	0.39	-8.45	3.36
	3	1	-10.46^*^	2.97	<0.01	-16.37	-4.55
		2	-0.63	2.97	0.83	-6.53	5.28
		4	-3.17	3.01	0.29	-9.14	2.80
	4	1	-7.29^*^	2.97	0.02	-13.19	-1.38
		2	2.55	2.97	0.39	-3.36	8.45
		3	3.17	3.01	0.29	-2.80	9.14

*The mean difference is significant at the 0.05 level.

**Table 10:** Descriptive Statistics of the difference of pre- and post-video tests across the four groups.

		Mean	Std. error
systolic blood pressure (SBP)	grassland	-12.54	3.43
	shrubs	-22.17	2.27
	grassland and trees	-22.96	2.2
	shrubs and trees	-18.61	2.37
diastolic blood pressure (DBP)	grassland	-11.58	2.21
	shrubs	-21.42	2.2
	grassland and trees	-22.04	1.8
	shrubs and trees	-18.87	2.16

## 4 Discussion

The purpose of our study was to investigate the extent to which vegetation design along UGW impacts people’s mental health. Ninety-four college students participated in the controlled experiment. We randomly assigned them to one of the four groups – grassland group, shrubs group, grassland and trees group, and shrubs and trees group. The results indicate that virtual exposure to UGW has a significant influence on mental health. Specifically, participants’ stress levels increased after the TSST and decreased following the video intervention. Virtual exposure to UGW also improved participants’ attentional functioning, which had been fatigued by the TSST. Additionally, UGW with different vegetation designs have varying effects on stress reduction. VR headsets can provide restorative experience for urban residents who have limited access to nature.

### 4.1 Benefits of urban greenways in promoting mental health

The primary focus of our study is to recognize the potential impacts of virtual exposure to UGW with different vegetation designs on stress reduction and mental health. The results of the repeated-measures ANOVA indicate notable variations in stress and attention indicators during the experiment, suggesting a trend towards the effectiveness of the video intervention in reducing stress and relieving mental fatigue. Both objective and subjective measures of mental health indicators tentatively point towards restorative effects associated with virtual exposure to UGW. The brief five-minute video, designed to provide an an immersive experience of UGW, appears to offer participants an initial glimpse into the potential benefits for mental health.

UGW as an important category of UGI has been receiving increasing attention because of the potential in promoting public health. While most of previous research focuses on the physiological benefits of exposure to UGI, researchers have started to recognize the psychological benefits of UGI as well (8, 9, 34). These studies add to our understanding of UGW as an essential type of UGI can also promote mental health, which is consistent with accumulative evidence of other UGI, demonstrating exposure to UGI can promote stress reduction (58) and attentional functioning (21, 26) and get more psychological benefits (38). For example, Chang, Tsou (34) established a structural equation model to investigate the relationship between UGW and happiness, which showed that better quality of greenway environment was associated with lower perceived stress levels, and higher levels of happiness among the elderly. In another study of multiple UGW in the United States, researchers demonstrate that UGW can promote physical activity, which eventually contributes to improved mental health (9). By quantifying the relationship between accessibility to the greenway and health outcomes, Xie, Lu (8) emphasized the importance of improving access to UGW as a strategy to promote public health. These studies all uphold that the intervention of UGW is an effective strategy to promote mental health. In line with this research, our findings provide substantial evidence supporting the restorative effects of UGW, highlighting their significance in reducing acute stress and restoring mental fatigue.

Our finding that a landscaped urban trail within the urban fabric efficiently promotes mental health underscores the importance of integrating UGWs into the built environment. Placing a greenway on the outskirts of a city reflects a strategic response to spatial conflicts in high-density urban areas, where space is often at a premium. To ensure that urban residents have access to UGW and the mental health benefits associated with it, policies should promote the incorporation of this form of UGI into urban planning decisions. Balancing spatial conflicts, ensuring accessibility, and integrating UGW within the urban fabric should be key considerations in shaping policies that support the mental wellbeings of urban residents. Moreover, UGW becomes a vital asset for urban policies focused on public wellbeings. It highlights the need for comprehensive strategies that prioritize UGI, recognizing its role in promoting mental health and enhancing the overall quality of life for urban residents.

The subjective and objective measures of stress and attention increase the internal validity of our research. Moreover, it is important to acknowledge that we did not include a control group with no vegetation along UGW in our research design because of two reasons. First, the urban greenways we studied all have vegetation on both sides. Actually, excellent natural environment surrounds these UGW. Second, prior studies have consistently shown that built environments with vegetation are better for people’s mental health than built environments without vegetation (21, 26, 58, 59). Therefore, we only included four types of vegetation design to explore the variation between groups in promoting mental health, which corresponds with our research objectives.

Furthermore, our study found that TSST increased participants’ objective stress levels, but their subjective stress remained steady from the baseline measure to the pre-video measure. This is because subjective stress evaluated by POMS assesses participants’ state of the past week. The transient effect of TSST may not be shown by the POMS scale. However, we have also noted that the video intervention decreased both objective and subjective stress indicators, providing further evidence that exposure to greenways can effectively reduce stress. Specifically, POMS measured at the post-video point is significantly different from that of the baseline and pre-video. The significant variations in POMS illustrate the great impact of video intervention on stress reduction.

### 4.2 Different effects on stress reduction between groups

In this randomized controlled experiment, we have explored between-group difference in promoting mental health by virtual exposure to four types of UGW. Our results indicate significant differences in SBP and DBP of the pre-video and post-video differences. Specifically, participants in the grassland group exhibited poorer stress reduction compared to those in the shrubs group as well as the grassland and trees group. Following previous research, it implies grassland, which is poor at supporting biodiversity (42), is negatively associated with psychological restoration. The other two groups – shrubs, and grassland and trees have a more diverse level of landscape. As we can see, the shrubs group include flowers, which are believed to improve the aesthetic qualities and structural diversity of landscapes (60). Meanwhile, many studies have already demonstrated the restorative effects of tree canopies (5, 61).

Therefore, our finding suggests that exposure to different types of UGW with varying levels of landscape heterogeneity may have shifting effects on stress reduction. Landscape heterogeneity – a combination of diversity and spatial arrangement, is a very promising measure of UGI (62), which may elucidate the relationship demonstrated by previous research between exposure to UGI and psychological restoration (21, 42). In another study of vegetation heterogeneity and health outcomes, researchers also illustrate that diversity in vegetation design contributes to better health outcomes (20). In line with these studies, our findings also suggest that increasing vegetation diversity can enhance the effect on stress reduction.

It is interesting to notice the outcomes of the shrubs and trees group, of which participants did not achieve better restorative effects than their counterparts in the grassland and trees group. This is in line with our previous study of the restorative effects of different UGI. Adding understory vegetation with diversity, such as bioswales or shrubs to trees seems to cut down the beneficial effects of trees (21). Besides the difference in the absence or presence of flowers, the other difference between the group of grassland and trees and the group of shrubs and trees are mainly in the height of the understory vegetation. According to the Prospect-Refuge theory (63), people have an innate preference for environments that are open, expansive and visually stimulating that provide unobstructed views of surroundings. For example, Kaplan and Kaplan (64) argue that an enclosed space can evoke a feeling of safety or relaxation while a view from that place can add levels of stimulation and excitement. In our study, both the group of shrubs and the group of grassland and trees are assumed to offer the sense of enclosure, as well as provide a view towards outside. However, a relatively strong sense of enclosure combined with a lack of visibility – inferred for the group of shrubs and trees – may diminish the stress-reduction effects.

### 4.3 Limitations and future research

In this study, we have employed the VR headset to provide participants with an immersive experience of UGW. It is worth noting that the representation of UGW in our study might not encapsulate the full spectrum of UGW characteristics found in diverse urban settings. UGW can vary significantly in terms of size, design, and surrounding infrastructure across different regions. Therefore, the generalizability of our findings to all urban greenways should be approached with caution. Future research should aim to include a more diverse range of UGW characteristics to enhance the external validity of our findings and better inform the broader applicability of our results to urban planning and mental health interventions.

Previous studies have demonstrated VR is an effective way to bring nature and its restorative benefits to people who have limited access to it in daily life (65, 66). VR allows researchers to exert precise control over experimental conditions. In our study, we have manipulated and customized the virtual environments – UGW with different vegetation design, to create controlled experimental scenarios. This level of control enhances the internal validity of our research, ensuring that results are more directly attributable to the manipulated variables. Moreover, the use of VR in our study highlights its wide-ranging potential in the fields of public health and medicine. With the ongoing progression of technology, additional investigation and scrutiny into the potential of VR in healthcare environments may yield inventive and efficacious interventions. For example, future research could delve into the integration of VR within therapeutic contexts, thereby providing immersive and controlled environments to facilitate mental health interventions. Incorporating VR-based relaxation programs in healthcare facilities could contribute to a more restorative experience for people, potentially benefiting their overall wellbeings.

Moreover, real-world experiences engage multiple senses simultaneously, providing a richer and more multisensory environment than VR exposure does. Built environments often include buildings and other man-made structures. People can also perceive the physical world through visual, auditory, tactile, olfactory, and gustatory senses. In contrast, VR exposure mainly provides visual stimuli in our study. Future research may explore how to incorporate other sensory modalities and man-made variables (e.g. building background) that represent local characteristics to the VR exposure. It is important to acknowledge the preliminary nature of our study and that further research is needed to draw conclusive insights into the extent of the restorative effects by exposure to UGW. Additionally, participants of our study are university students who may have different daily routines, which can impact their mental health status. Even though we randomly assigned participants to one of the four groups to increase the validity of our study, future research might take a record of participants’ daily activities over a period or in a task-unconstrained condition so that researchers may understand the impacts of daily exposure in real-world settings on mental health.

Our study has primarily focused on university students as subjects, prompting future research into exploring different demographic groups that also derive restorative benefits from UGWs. Previous research has already managed to understand how diverse demographics interact with and perceive UGI (67), and the variance in availability of UGI across demographic groups (68). Vulnerable population groups are commonly believed to have a greater demand for health benefits provided by public UGI. Various demographic groups have been examined in empirical research to ascertain the health benefits associated with UGI exposure. These groups consist of children and adolescents (69, 70), the elderly (71), individuals from low socioeconomic status (72), women who are pregnant (73) and children with Attention Deficit/Hyperactivity Disorder (70). These findings have important implications for nature-based health interventions in urban planning and landscape design. Moreover, understanding the nuanced ways in which specific demographics may derive optimal advantages from the presence of UGWs can aid in the allocation of natural resources to areas with the greatest demands. By delving into these demographic nuances (such as age, socioeconomic status, etc.), future research can guide more targeted and efficient research efforts, ensuring that the benefits of UGWs are maximized for a broad spectrum of the population.

While researchers, administrators and many stakeholders have started to realize the benefits of UGW in promoting mental health, we still need more empirical evidence to understand the effects of different vegetation design along the UGW. Previous studies have demonstrated vegetation types as a significant feature that correlates visual aesthetics and landscape types (74), which play an important role determining the visual aesthetic quality (VAQ) of landscape (75). Specifically, strong color contrast and mixed use of evergreen and deciduous vegetation can increase VAQ effectively (76). Meanwhile, increasing vegetation coverage would also contribute to VAQ and people’s preference in practice (77), which promote overall wellbeing eventually (25). As landscape aesthetics are associated with the efficiency of creating restorative environments (78), the visual aesthetics of urban landscape, especially the restorative attributes of nature-based stimuli are expected to be further explored. In our study, the results of the group of shrubs and trees suggest that adding shrubs to trees and adding trees to shrubs can both reduce the effects of stress reduction. We argue that the sensation of enclosure created by vegetation of varying heights contributes to variation in restorative outcomes. As there are only four types of vegetation designs in our research, researchers may continue to explore the impacts of landscape heterogeneity in biodiversity and configuration on health outcomes. By verifying the health outcomes of visual aesthetics and color composition influenced by various vegetation designs along UGW, we can apply these findings to the construction of UGW in the future and achieve increasing health benefits.

## 5 Conclusion

This study provides insight into the vegetation design along UGW to promote human health. Despite the boom in the construction of UGW in China in recent years, empirical evidence of landscape design along greenways on mental health remains scarce. Therefore, four different types of UGW were manipulated in this study, with the aim of investigating the effects of different vegetation design along UGW on mental health.

In conclusion, our study suggests that UGW with vegetation on both sides has a positive effect on stress reduction and attention restoration. Specifically, the effect of stress reduction varied depending on the types of vegetation along UGW. Participants in the group of shrubs and in the group of grassland and trees received more health benefits in terms of stress reduction compared to their counterparts in the grassland group. Overall, grassland is not a supportive type of vegetation for promoting mental health. Our findings presented here can provide evidence for city administrators, designers and other stakeholders interested in urban greenway initiatives, providing them confidence in creating healthier greenways for the public.

## Data availability statement

The original contributions presented in the study are included in the article/supplementary materials, further inquiries can be directed to the corresponding author/s.

## Ethics statement

The studies involving humans were approved by The ethics committee of Xihua University. The studies were conducted in accordance with the local legislation and institutional requirements. The participants provided their written informed consent to participate in this study. Written informed consent was obtained from the participants for the publication of any potentially identifiable images or data included in this article.

## Author contributions

XJ: Conceptualization, Data curation, Formal analysis, Supervision, Validation, Writing – review & editing. XWa: Conceptualization, Data curation, Formal analysis, Investigation, Methodology, Project administration, Software, Visualization, Writing – original draft. LH: Investigation, Validation, Writing – original draft. QG: Investigation, Validation, Writing – original draft. XWe: Investigation, Validation, Writing – original draft. MX: Investigation, Software, Visualization, Writing – original draft. WS: Conceptualization, Validation, Writing – review & editing.
